# Review of ultrasonic irrigation in endodontics: increasing action of irrigating solutions

**DOI:** 10.4317/medoral.17621

**Published:** 2011-12-06

**Authors:** Sandra Mozo, Carmen Llena, Leopoldo Forner

**Affiliations:** 1 Postgraduate Endodontics Program. Department of Stomatology, University of Valencia, Spain; 2 Professor. Department of Stomatology, University of Valencia, Spain. Primary Care Dentist, Department 9, Valencian Health Service, Spain; 3 Professor. Department of Stomatology, University of Valencia. Spain

## Abstract

Introduction: Effective irrigant delivery and agitation are prerequisites for successful endodontic treatment. Ultrasonic irrigation can be performed with or without simultaneous ultrasonic instrumentation. Existing literature reveals that ultrasonic irrigation may have a very positive effect on chemical, biological and physical debridement of the root canal system as investigated in many in vitro studies. Objective: The purpose of this review article was to summarize and discuss the available information concerning ultrasonic irrigation in endodontics. Methods: This article presents an overview of ultrasonic irrigation methods and their debridement efficacy. In this paper the relevant literature on passive ultrasonic irrigation is reviewed. Information from original scientific papers or reviews listed in MEDLINE and Cochrane were included in the review. Results: The use of ultrasound in the irrigation procedure results in improved canal cleanliness, better irrigant transfer to the canal system, soft tissue debridement, and removal of smear layer and bacteria. There are many in vitro studies, but there is a need to standardize protocols, and correlate the clinical efficacy of ultrasonic devices with improved treatment outcomes. Understanding the basis of ultrasonic irrigation is fundamental for clinicians and researchers to improve the design and use of ultrasonic irrigation.

** Key words:**Ultrasonic irrigation, ultrasound, smear layer, endodontics.

## Introduction

Removal of the remains of vital and necrotic pulp tissue, microorganisms and microbial toxins from the root canal system is essential for successful endodontic treatment (1).

Irrigating solutions act mainly as lubricant and cleaning agent during biomechanical treatment, removing microoganisms, products asociated to tissue degeneration and organic and inorganic remains, guaranteeing elimination of contaminated dentin and permeability of the canal throughout its length (2). Effective action is achieved by ensuring that irrigants come into direct contact with all canal walls, particularly in the more apical portion.

At present, no single irrigant combines all the ideal characteristics, even when they are used with a lower pH, increased temperature or added surfactants to increase their wetting efficacy (3,4). No single irrigant has demonstrated an ability to dissolve organic pulp material and demineralise the calcified organic portion of canal walls.

In practice, current endodontic treatment uses two irrigants, sodium hypochlorite (NaOCl), alone or in combination with ethyl-enediaminetetraacetic acid (EDTA) or chlorhexidine (3).

Throughout the history of endodontics, ongoing efforts have been made to develop more effective systems to send and agitate irrigant solutions in the canal system. These systems can be divided into two broad categories of manual and mechanical agitation techniques. Machine-assisted procedures include using rotary brushes, simultaneous irrigation with rotary instrumentation of the canal, pressure alternation devices and sonic and ultrasonic systems. All of them appear to improve canal cleaning in comparison to conventional syringe and needle irrigation (5).

This study proposes a review of the use of ultrasound as a technique for agitating irrigant solutions in root canals and its advantages and limitations in relation to conventional irrigation procedures.

## Material and Methods

This review is based on a search of the MEDLINE and Cochrane databases using the terms: ultrasonic irrigation AND (ultrasound OR endodontics OR smear layer). Publications from 1990 to 2010 were analysed.

A total of 159 papers were retrieved. Literature reviews and experimental trials related to ultrasound irrigation in endodontics were used, providing a total of 28 publications for this study. Three historical references have been added.

## Nature of ultrasound

Ultrasound is a vibration or acoustic wave of the same nature as sound but at a frequency higher than the highest frequency perceptible to the human ear (approximately 20,000 Hz).

There are two basic methods for producing ultrasound. Firstly, by magnetostriction that converts electromagnetic energy into mechanical energy. Various strips of magnetostrictive metal in a hand-held piece are joined to a stable, alternating magnetic field producing vibrations as a result. The second method, based on the piezoelectric principle, uses a crystal which changes size when an electrical charge is applied. When the crystal deforms, it goes into mechanical oscillation without producing heat. Magnetostrictive units create figures of eight (elliptical movement), which is not ideal for endodontic use and another drawback with these units is that heat is generated, so adequate cooling is required. Piezoelectric units have some advantages over magnetostrictive units as they produce more cycles per second, 40 as against 24 kHz. The tips of these units work in a linear movement from back to front like a piston which is ideal for endodontic treatment (6).

One of the most important advantages of ultrasonic tips is that they do not rotate, thereby delivering safety and control while maintaining high cutting efficacy. Nodes and antinodes are produced throughout the length of an endosonic file activated by a 30kHz piezoelectric generator and so the file displacement amplitude does not increase linearly with increasing generator power (7). This finding applies in particular when hidden canals are permeabilized or when posts or fractured instruments are withdrawn. This movement is also ideal in endodontic surgery when a preparation for retrograde filling is created.

## Ultrasound applications in endodontic treatment

Ultrasound was first used in dentistry to prepare cavities. The concept of “Minimally Invasive Dentistry” and the desire for small-sized cavity preparations meant a new application of US for cavity preparation. However, it did not become popular until 1955, the year when a new application was introduced using US to remove calculus deposits and plaque from teeth surfaces. Despite the fact that US is used in dentistry for therapeutic and diagnostic purposes and also for cleaning instruments before sterilisation, the main use until recently has been for scraping and smoothing the root surfaces of teeth and root canal treatment (6,8).

Richman first introduced ultrasonic instrumentation to endodontics in 1957 for root canal therapy with Cavitron© as irrigation and obtained good results. However, ultrasonically activated K files were not used for preparing canals before filling until the study by Martin et al. (9). The term “endosonic” was coined by Martin and Cunningham (10) and was defined as the ultrasonic synergistic system of instrumentation and canal disinfection.

US in endodontic treatment has improved treatment quality in many aspects, including: access to root canal entry holes, cleaning, shaping and filling canals, eliminating obstructions and intracanal materials and endodontic surgery (6).

## Ultrasonic irrigation

The literature describes two types of ultrasonic irrigation. The first is the simultaneous combination of ultrasonic irrigation and instrumentation. The second type functions without simultaneous instrumentation and is known as passive ultrasonic irrigation (PUI) (11). The first one has been almost discarded in the clinical practice, because of the difficulty of controlling the cut of dentin and subsequently the final shape of the prepared canal, being present the possibility of making aberrant conformations. When ultrasonic-activated files are used, canal deviations, apical zips and radicular perforations can be present, especially in curved canals (12). Is therefore not considered as an alternative to conventional manual instrumentation (11,13,14).

The literature claims that it is more advantageous to apply ultrasound for passive irrigation (15,16). The term PUI was first used by Weller et al. (17) in 1980 to describe irrigation without simultaneous instrumentation. This non-cutting technology reduces the potential for creating aberrant shapes in the root canal system. During PUI, energy is transmitted from a file or smooth oscillating wire to the irrigant by means of ultrasonic waves that induce two physical phenomena: stream and cavitation of the irrigant solution. The acoustic stream can be defined as a rapid movement of the fluid in a circular or vortex shape around the vibrating file. Cavitation is defined as the creation of steam bubbles or the expansion, contraction and/or distortion of preexisting bubbles in a liquid (13).

During the last decade, numerous successful devices have appeared for agitating irrigant solutions, that provide various irrigant transfer mechanisms, elimination of soft tissue and also, depending on the treatment philosophy, elimination of the smear layer. In comparison to sonic irrigation, ultrasonic irrigation has proved to be more powerful and able to eliminate more debris, and so it is claimed that passive ultrasonic irrigation is significantly more efficient than sonic activation (8). However, both techniques may clean the canal system to a similar degree when sonic irrigation is applied for a longer time (5,13,18). The capacity of irrigating solutions with good wetting ability to dissolve tissue can be improved by ultrasound if the pulp tissue debris and/or the smear layer are thoroughly moistened by the solution and it is subjected to ultrasonic agitation (4).

As a complement to various irrigant solutions, ultrasounds contributes to the elimination of the smear layer (2,6), appearing less effective in improving EDTA activity (11), however, it has been stated that the association with EDTA improves cleaning of the root canal wall after preparation of the space for fitting a post in endodontically treated teeth, especially in the apical part of the space housing the post (19).

Of all the known irrigants, none have been as effective as 5.25% sodium hypochlorite solution (15). Irrigation with NaOCI com-bined with ultrasound or a wave vibration system has the greatest antibacterial effect. Use of this combination improves the exchange of substances in the canal, permits heating of the irrigating substance, eliminates dentin debris and part of the waste layer, thereby achieving greater cleaning effect (3). In general, the literature recommends between 30 seconds and 3 minutes for NaOCl irrigation, although there is no defined consensus on the exact length of time. Shorter passive irrigation makes it easier to keep the file in the centre of the canal and therefore prevents it from touching the walls and creating aberrant forms (16).

Other systems, as RinsEndo, with a hydrodynamic activation based on the pressure-suction technology, has recently been introduced to the market, this device has not showed to be more effective than PUI in removing root canal (20).

The effectiveness of irrigation depends on stream action and the chemical ability of the irrigants to dissolve tissue (5). With syringes, stream action is relatively weak and depends on both root canal anatomy and the depth of the needle according to its diameter. It has been shown that irrigants can only progress 1 mm beyond the tip of the needle. Increased volume does not significantly improve cleaning action or detritus elimination (8).

The only effective way of cleaning the canal system is by moving the irrigant solution as the network of side canals cannot be cleaned mechanically (21). US are a useful complement for cleaning difficult anatomic areas. It has been shown that effective cleaning of the root canal system is directly associated with an irrigant used in combination with ultrasound vibration which generates continuous movement in the irrigant (18).

When files are passively activated with ultrasound energy, the acoustic cut is sufficient to produce significantly cleaner canals in comparison to the use of manual instrumentation alone. Small vibrating files together with high ultrasonic power has been recommended (16) as finer files pose less risk of deforming the canal. Ultrasonic vibration can also be effective when it touches the handle of a manual file inserted in the canal. The manual file will transmit the vibrations to the irrigant in the canal but there is a greater risk of touching and deforming the walls.

In canals that are wider in the apical region, debridement and disinfection are improved. Thorough cleaning of most apical parts for any preparation is difficult (22,16). The use of finer needles (30G caliber) may facilitate direct access to the apical region. Although conclusive evidence is lacking, the introduction of fine irrigation needles with a safety tip placed at the working length or 1 mm shorter can improve irrigant effectiveness (23).

It has been shown that in canal irregularities and oval-shaped canals, after syringe irrigation large amounts of dentine debris remain (2,24). During ultrasound irrigation, the oscillation of the file in the vicinity of canal irregularities can also eliminate more debris from these difficult to reach locations (13,16,21).

The efficacy of manual syringe irrigation in narrow canals has been questioned by various researchers. Narrow canals may also compromise the efficacy of ultrasonic irrigation. When sonic or ultrasonic files are used in small, curved canals, their free vibratory movement is restricted and possibly also their cleaning efficacy. Therefore, in small diameter roots, irrigant solutions have difficulty reaching the apex and therefore are less influenced by activated irrigation (25), while ultrasound irrigation is more effective in wide canals (10). Therefore, it seems important to apply the ultrasound instrument after completing preparation of the root canal. Also, free oscillation of the instrument will cause more ultrasonic effects in the irrigant solution than an oscillation forced against canal walls (5). The use of a smooth wire during ultrasonic irrigation in vitro seems to be as effective as a K file in eliminating debris (14,16).

Two flushing methods can be used during PUI, continuous or intermittent flush of the irrigant (8). The continuous flush technique provides an uninterrupted supply of fresh irrigation solution in the root canal. According to some authors (5) this technique can provide more effective results and reduce the time required for ultrasonic irrigation. This is due to the fact that chloride (responsible for dissolving the organic tissues and NaOCl’s antibacterial property) is unstable and quickly consumed during the first part of tissue dilution, probably within two minutes. In the intermittent flush technique the irrigant is injected in the root canal with a syringe, the irrigant solution is then activated with an oscillating ultrasonic instrument and the canal is filled several times after each activation cycle. The amount of irrigant flushed through the apical region of the canal can be controlled by the depth of penetration of the syringe and the volume of irrigant. This degree of control is not possible with continuous flush. Both flush methods have proved to be equally effective in removing dentin debris from the root canal in an ex vivo model when irrigation time was set at three minutes (13,15).

## Discussion

There is a general consensus that PUI is more effective than conventional syringe and needle irrigation in eliminating pulp tissue and dentin debris. This difference may be due to the fact that ultrasound creates a higher speed and flow volume of the irrigant in the canal during irrigation, thereby eliminating more debris, producing less apical packing, better access of the chemical product to accessory canals and even the flush effect produced by ultrasound but not manual irrigation (14). Nevetherless, a PUI procedure is needed because the ultrasonic file can move freely in the canal (26), avoiding dentinal injuries and the corresponding complications as perforations or shape irregularities.

With regard to the elimination of the smear layer, the accumulated evidence indicates that PUI with water as irrigant does not eliminate the smear layer (13), but a complete elimination of the smear layer using PUI with 3% NaOCl has been reported (22). These results were confirmed in subsequent studies using different concentrations of NaOCl (25). Therefore, an effective irrigant must be combined with the use of a technique that facilitates access to the difficult areas of the canal. Other studies show less conclusive results for the efficacy of ultrasonic irrigation in removing the smear layer. Despite the fact that PUI proved to be significantly better than needle irrigation, a study (27) reports that the smear layer was not completely eliminated when using PUI with 1% NaOCl for 10 seconds. EDTA has been related to better smear layer removal during the use sonically activated irrigation (28) and PUI (29).

Numerous researchers have shown that the use of PUI after manual and rotary instrumentation significantly reduces the number of bacteria, achieving significantly better results than needle and syringe irrigation (2-4,6,8). These positive results could be due to two main factors, firstly high power ultrasound produces a disagglomeration of bacteria biofilms in the root canal by the action of the acoustic current. The deconstruction of bacterial biofilms gives rise to planktonic bacteria that are more susceptible to the bactericidal activity of NaOCl. Cavitation may also produce a temporary weakening of the cell membrane making bacteria more permeable to NaOCl (24).

However, some studies show that although the number of surviving colonies is reduced when ultrasonic activation is used, no technique is able to ensure complete disinfection (30).

Some authors maintain that the best moment to apply irrigants with PUI to improve flow action is in the initial phase of endodontic treatment so that the irrigant can be sprayed into the pulp chamber. In this phase ultrasound has the advantage of enabling the irrigation medium to flow towards the apical third using fine files. Nevertheless, most authors state that the best moment for ultrasound activation of the irrigant is after shaping the root system, in the final phase of irrigation as this enables the needle to be introduced throughout the working length thereby increasing irrigation efficacy, since as some authors show, the factors that favour irrigation are: needle depth, proportion of the radius of the root canal and the irrigation needle and diameter to which the channel is prepared (14).

## Conclusions

After the literature review it can be concluded that the most advisable technique for clinical use is to supplement conventional syringe irrigation in the initial phase of canal preparation with a final phase of intermittent passive ultrasonic irrigation after sufficiently preparing the root canal system. The combination of conventional irrigation together with ultrasonic irrigation facilitates the procedure and improves the elimination of bacteria and the smear layer throughout the canal system thereby contributing to higher success rates for endodontic treatment.
